# Clinical and laboratory characteristics of ocular syphilis: a new face in the era of HIV co-infection

**DOI:** 10.1186/s12348-015-0056-x

**Published:** 2015-08-22

**Authors:** Sun Young Lee, Vincent Cheng, Damien Rodger, Narsing Rao

**Affiliations:** Department of Ophthalmology, USC Eye Institute, Keck School of Medicine of the University of Southern California, 1450 San Pablo St., Suite 4000, Los Angeles, CA 90033 USA

**Keywords:** Ocular syphilis, HIV, RPR titer

## Abstract

**Background:**

Ocular syphilis is reemerging as an important cause of uveitis in the new era of common co-infection with HIV. This study will reveal the clinical and laboratory characteristics in the group of individuals co-infected with ocular syphilis and HIV compared with HIV-negative individuals. In this retrospective observational case series, medical records of patients diagnosed with ocular syphilis with serologic support from 2008 to 2014 were reviewed. Ocular and systemic manifestation and laboratory profiles were reviewed.

**Results:**

Twenty-nine eyes of 16 consecutive patients (10 HIV-positive and 6 HIV-negative) were included. All patients were males, and mean age of onset for ocular syphilis was 43 (mean 42.65 ± 13.13). In both HIV-positive and HIV-negative groups, ocular manifestations of syphilis were variable including anterior uveitis (4 eyes), posterior uveitis (8 eyes), panuveitis (13 eyes), and isolated papillitis (4 eyes). In HIV-positive patients, panuveitis was the most common feature (12/18 eyes, 67 %) and serum rapid plasma reagin (RPR) titers were significantly higher (range 1:64–1:16,348; mean 1:768; *p* = 0.018) than in HIV-negative patients. Upon the diagnosis of ocular syphilis in HIV-positive patients, HIV-1 viral load was high (median 206,887 copies/ml) and CD4 cell count ranged from 127 to 535 cells/ml (mean 237 ± 142; median 137). Regardless of HIV status, cerebrospinal fluid (CSF) exam was frequently abnormal: positive CSF fluorescent treponemal antibody absorption (FTA-ABS) or Venereal Disease Research Laboratory (VDRL) test results in seven patients or either elevated CSF WBC count or elevated CSF protein in six patients.

**Conclusions:**

Our results reveal that the patients with ocular syphilis with high serum RPR titers may have concomitant HIV infection requiring further testing for HIV status and ocular syphilis is likely associated with the central nervous system involvement and therefore needs to be managed according to the treatment recommendations for neurosyphilis.

## Background

Syphilis is a multi-systemic infection caused by the spirochete *Treponema pallidum*. Since the introduction of antibiotics, notably penicillin, the incidence of syphilis decreased dramatically and reached its lowest recorded level in the year 2000 [1]. However, the number of syphilis cases has steadily increased since then. This increase was attributed to syphilis outbreaks in multiple regions of the USA including Southern California [2]. In the recent outbreaks, high rates of HIV co-infection were documented, ranging from 20 to 70 % [1–4]. Kaiser Permanente, Northern California, reported syphilis incidence stood at 62.3 per 1000 person-years in the HIV group vs 0.8 per 1000 person-years in the non-HIV group. Statistical analysis adjusted for age, gender, and HIV status determined that HIV-positive individuals had an 86 times higher syphilis risk [5].

Reportedly, neurosyphilis, one of the most devastating complications of syphilis, is now more commonly seen in patients infected with HIV than was previously reported. Furthermore, neurological manifestations in syphilis have changed; there is now a higher prevalence and faster progress to the early forms of neurosyphilis such as acute meningitis and meningovasculitis than the later form such as paretic neurosyphilis or tabes dorsalis [6, 7]. In addition, reduced sensitivity and specificity of syphilis serology and cerebrospinal fluid (CSF) tests making the diagnosis of syphilis more challenging were reported in HIV co-infected patients [8, 9].

Ocular inflammation was observed up to one third of all neurosyphilis patients [10]. Although numerous studies on ocular syphilis have been reported, there are limited data about HIV-related risk factors such as HIV viral load or CD4+ cell count as they relate to syphilis serology and CSF laboratory findings. This is particularly important because ocular syphilis can present in the absence of neurological manifestations [10]. As such, in this new era of reemergence of syphilis associated with HIV co-infection, the present study aims to determine the clinical and laboratory characteristics of ocular syphilis in HIV-positive patients compared to HIV-negative patients.

## Methods

### Data collection

The clinical, laboratory, and ocular image records of patients with ocular syphilis from January 2008 through April 2014 at the Los Angeles County Medical Center were retrospectively reviewed. Institutional review board of the University of Southern California approved this study. Patients with various ocular manifestations with positive non-treponemal rapid plasma reagin (RPR) and treponemal fluorescent treponemal antibody absorption (FTA-ABS) assay serologies were included. Historical details included duration of ocular symptoms, age, ethnicity, gender, and sexual orientation. All patients underwent a complete physical examination and HIV screening regardless of known previous history. Serologic test results, CSF analyses, and fluorescein angiography images were also examined. In addition, CNS imaging was also performed. All of these findings were compared between HIV-positive patients and HIV-negative patients. Type of uveitis was classified into anterior uveitis, intermediate uveitis, posterior uveitis, and panuveitis following the Standardization of Uveitis Nomenclature (SUN) Working Group schema.

### Statistical analysis

The significance of syphilis serology findings on serum and CSF was determined using univariate analysis. Serum RPR titer was maintained as a continuous variable, and the Mann-Whitney *U* test was used to examine the association between HIV status and RPR titer. However, CSF serology was reported as a dichotomous variable; patients with negative results for both the FTA-ABS and Venereal Disease Research Laboratory (VDRL) tests were considered CSF-negative, while patients with either a positive FTA-ABS or positive VDRL were considered CSF-positive. Fisher’s exact test was then used to examine the association between HIV status and CSF status.

Data were managed and analyzed using SPSS version 17.0 (IBM Corporation, Armonk, NY). All statistical tests were 2-sided with *p* < 0.050 considered statistically significant.

## Results

### Demographics

**Fig. 1:**
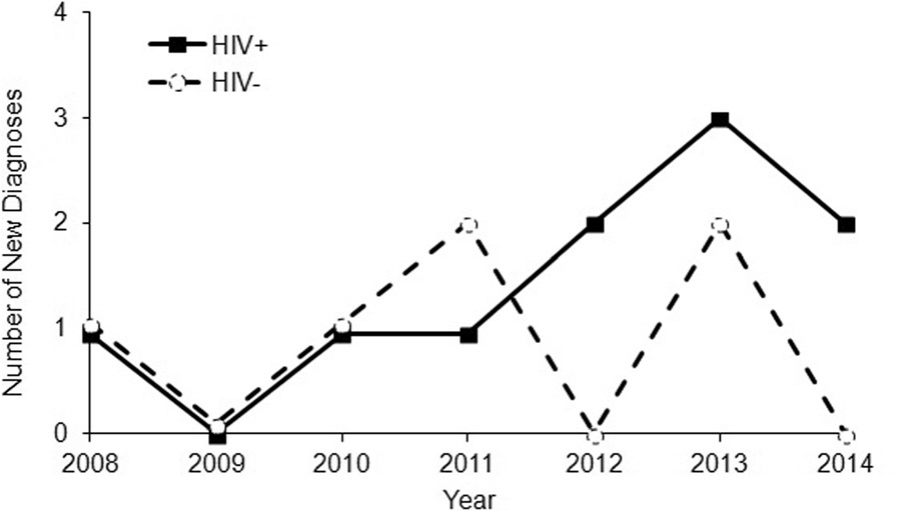
Newly diagnosed ocular syphilis patients by year

Sixteen consecutive patients (10 HIV-positive vs 6 HIV-negative) with 29 involved eyes were diagnosed with ocular syphilis. Among these patients, the number of HIV-positive patients presenting with ocular syphilis has increased over 2012 to 2014 from its level from 2008 to 2011 (Fig. 1). All patients were males, and mean age of onset was 43 (mean 42.65 ± 13.13). Seven patients out of 10 HIV-positive patients were men who have sex with men (MSM).

### Ocular manifestation

Regardless of HIV status, ocular findings of ocular syphilis were variable including anterior uveitis (4 eyes), posterior uveitis (8 eyes), panuveitis (13 eyes), and isolated papillitis (4 eyes). However, panuveitis was the most common diagnosis (12/18 eyes, 67 %) in HIV-positive patients whereas posterior uveitis was the predominant diagnosis (6/11 eyes, 55 %) in HIV-negative patients (Table 2). In HIV-positive patients, either isolated or combined disc swelling was noted in 14 eyes in contrast to only 1 eye in HIV-negative patients.

All patients were treated with intravenous (IV) benzyl penicillin 18–24 MU/day for 10 to 14 days. Most of the patients responded to intravenous penicillin with relatively good visual outcome (Table 2).

### Systemic manifestations

All patients were sent for complete neurologic work-up, and 15 of 16 patients underwent lumbar puncture (LP) for CSF analyses. Among 10 HIV-positive patients, six patients (cases 2, 3, 4, 5, 6, and 9) were positive for either FTA-ABS or VDRL on CSF serology. Three patients (cases 2, 5, and 6) presented with a maculopapular skin rash, alopecia of the scalp, and/or oral ulcers, and one patient (case 7) presented with headache and new onset tinnitus. One patient (case 2) from the HIV-positive group showed cerebral volume loss on MRI. Among six HIV-negative patients, one patient (case 12) presented with oculomotor and facial nerve palsies and one another patient (case 16) presented with tabes dorsalis, with positive VDRL on CSF exam. Two other patients (cases 12 and 15) from the HIV-negative group showed diffuse cerebral volume loss on either brain MRI or CT studies although no other focal neurologic sign was observed. Inflammatory changes in the meninges were not observed radiographically in any of these patients.

### Laboratory profiles

**Fig. 2:**
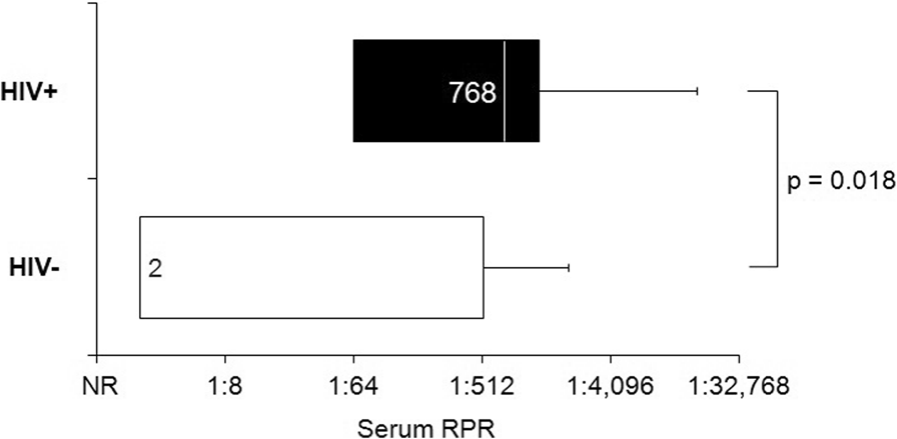
Serum RPR titers at diagnosis by HIV status

All patients were positive for both the RPR and FTA-ABS serology tests. Significantly, higher serum RPR titers were found in HIV-positive patients (range 1:64–1:16,348) than in HIV-negative patients (range 1:2–1:8, except one patient with 1:2048) (*p* = 0.018) (Fig. 2). The HIV-negative patient with high serum RPR titer (case 16) presented with tabes dorsalis in addition to isolated bilateral papillitis. In all of the HIV-positive patients and two out of four HIV-negative patients who had follow-up serum RPR titers, the elevated non-treponemal titers were decreased following IV penicillin treatment (Table 2).

In HIV-positive patients, 9 out of 10 patients underwent LP and CSF FTA-ABS or VDRL was positive in 6 patients (2 positive CSF VDRL and 4 positive CSF FTA-ABS) and CSF WBC count was elevated in 1 of the remaining 3 patients (>20 cells/mm^3^) [11]. All of the HIV-negative patients underwent LP, and in one of the six patients, CSF VDRL was positive. On the other hand, in five out of the six patients, either CSF protein (>40 mg/dl) or CSF WBC count (>5 cells/mm^3^) was elevated [12]. Due to the low numbers of patients in the study, however, this trend of more frequent positive CSF FTA-ABS or VDRL being found in HIV-positive patients (60 % in HIV-positive vs 16 % in HIV-negative) was not statistically significant (*p* = 0.145) (Table 1).

**Table 1:** Syphilis serology test of serum and CSF in HIV-positive and HIV-negative patients

	Age/gender/race	HIV	Serology	CSF P^a^; C^b^	CD4^c^	HIV-1 viral load^d^	Last RPR
1	40/M/B	+	RPR+ (1:64); FTA-ABS+	NR P:30; W:21	535	24,561	NR (5 years)
2	43/M/H	+	RPR+ (1:1024); FTA-ABS+	FTA-ABS+ P:46; W:4	137	>5,000,000	1:64 (15 months)
3	20/M/H	+	RPR+ (1:2048); FTA-ABS+	FTA-ABS+ P:97; W:189	376	189,089	1:16 (10 months)
4	28/M/W	+	RPR+ (1:16,384); FTA-ABS+	VDRL+ P:36; W:7	127	496,559	1:16 (1 year)
5	27/M/H	+	RPR+ (1:512); FTA-ABS+	VDRL+ P:79; W:18	134	NA	NA
6	48/M/W	+	RPR+ (1:64); FTA-ABS+	FTA-ABS+ P:43; W:37	124	224,685	NA
7	49/M/H	+	RPR+ (1:64); FTA-ABS+	NR P:25; W:3	NA	NA	1:8 (4 months)
8	25/M/B	+	RPR+ (1:1024); FTA-ABS+	NR P:20; W:0	134	NA	NA
9	46/M/H	+	RPR+ (1:1024); FTA-ABS+	FTA-ABS+ P:23; W:1	371	117,779	1:64 (1 month)
10	50/M/H	+	RPR+ (1:256); FTA-ABS+	NA NA	201	NA	NA
11	59/M/H	−	RPR+ (1:2); FTA-ABS+	NR P:543; W:2			1:2 (1 month)
12	63/M/H	−	RPR+ (1:2); FTA-ABS+	NR P:63; W:1			NA
13	51/M/B	−	RPR+ (1:2); FTA-ABS+	NR P:48; W:0			NA
14	52/M/H	−	RPR+ (1:2); FTA-ABS+	NR P:50; W:3			NR
15	58/M/B	−	RPR+ (1:8); FTA-ABS+	NR P:20; W:1			1:8 (6 months)
16	55/M/O	−	RPR+ (1:2048); FTA-ABS+	VDRL+ P:86; W:6			1:128 (1 year)

*B* black, *W* white, *H* Hispanic, *M* male, *RPR* rapid plasma reagin, *FTA-ABS* fluorescent treponemal antibody absorption, *NR* non-reactive, *NA* not available

^a^CSF protein mg/dl

^b^CSF WBC count cell/ml

^c^cell/ml

^d^copies/ml

At the onset of ocular symptoms in HIV-positive patients, HIV-1 viral load ranged from 24,561 to >5,000,000 copies/ml (median 206,887; mean 1,008,779 ± 17,306) and CD4 cell count ranged from 127 to 535 cells/ml (mean 237 ± 142) (Table 1). Six of 10 HIV-positive patients were newly diagnosed with HIV during this time. Of the remaining four patients with previously diagnosed HIV, three of them (cases 7, 8, and 10) were on HAART treatment.

## Discussion

In our study, among 16 patients who were diagnosed with ocular syphilis, 10 patients were HIV-positive whereas 6 patients were HIV-negative. The yearly number of HIV-positive patients with ocular syphilis has increased over its 2008 to 2011 level in the years 2012–2014 (Fig. 1). Such an increase is also supported by a recent report from Centers for Disease Control and Prevention (CDC) revealing significantly increased primary and secondary syphilis cases since 2009 and a high rate of HIV co-infection [1]. The 2010 Early Syphilis Annual Surveillance Report for Los Angeles County reported that, among 516 newly diagnosed with early syphilis, 58 % of the cases were HIV infected [3]. Furthermore, in our study, among 10 HIV-positive patients, six of those patients were newly diagnosed with HIV during the assessment of their ocular syphilis. Similarly, Kunkel reported 24 ocular syphilis patients presented from 1998 to 2006. In their series, 11 patients were noted to be HIV-positive and 7 out of 11 patients were newly diagnosed with HIV [6]. Moreover, in a CDC report with 147 patients with neurosyphilis from four major cities from 2002 to 2004, neurosyphilis was the sentinel presentation of HIV in 49 patients (33 %) [13]. These results and our series suggest that HIV-positive patients may often present with ocular syphilis before the HIV status is known. There have been a number of hypotheses regarding increased dual infection of HIV and syphilis. Historically, syphilis has similar epidemiologic risk factors with HIV, particularly among MSM. In addition, HIV may modify the natural course of syphilis in these patients by modulating immunologic response to *T. pallidum* in a way that may increase the propensity of the disease to progress to neurosyphilis [14].

While syphilis may affect the eye in various ways, uveitis is the most common ocular presentation [7]. The diagnosis of ocular syphilis based on ophthalmic findings, however, is often challenging due to lack of pathognomonic findings. In our study, regardless of HIV status, ocular findings of syphilis were variable including anterior uveitis (4 eyes), posterior uveitis (8 eyes), panuveitis (13 eyes), and isolated papillitis (4 eyes). This implies that a high index of suspicion of syphilis is required on screening for etiologic diagnosis in various types of uveitis. For those 18 eyes in the HIV-positive group, the majority (67 %) had panuveitis whereas for those 11 eyes in the HIV-negative group, the majority (55 %) had posterior uveitis at initial presentation (Table 2). Such findings may suggest that intraocular inflammation could be more severe in HIV-positive patients since panuveitis involves the entire uveal tract whereas the posterior uveitis is limited to the choroid. Previously, Hughes reported that retinitis with panuveitis was the most common ocular presentation regardless of HIV status and the strong association between HIV co-infection and syphilitic retinitis (100 % HIV-positive vs 14 % in HIV-negative) suggesting that HIV infection may modulate the severity of ocular syphilis [15]. Tran also found a high frequency of posterior uveitis in the presence of HIV and syphilis co-infection [16]. Similarly to our study, both Hughes and Tran suggested that syphilitic ocular inflammation of syphilis in HIV-infected patients seems to diffuse [15, 16]. The latest British Ocular Syphilis Study (BOSS) also reported that HIV-positive patients had higher rates of panuveitis than HIV-negative patients, although there were no significant differences in presenting or posttreatment visual acuity between two groups [17].

**Table 2:** Clinical findings of ocular syphilis in HIV-positive and HIV-negative patients

	Initial VA	Type of uveitis	Treatment	Final VA	FU duration
1	OD 20/50	Posterior uveitis	IV PCN × 14 days	OD 20/40	8 years
	OS 20/30	Posterior uveitis		OS 20/40	
2	OD LP	Panuveitis	IV PCN × 14 days	OD 20/25	3 years
	OS 20/30	Panuveitis		OS 20/80	
3	OD 20/30	Panuveitis	IV PCN × 14 days	OD 20/70	2 years
	OS 20/40	Panuveitis	IM PCN × 3 weeks	OS 20/40	
4	OD 20/20	None	IV PCN × 14 days	OD 20/25	10 months
	OS 20/200	Panuveitis	IM PCN × 3 weeks	OS 4 ft/200	
5	OD 20/25	Anterior uveitis	IV PCN × 10 days	OD 20/30	1 months
	OS 20/40	Panuveitis		OS 20/40	
6	OD 20/150	Panuveitis	IV PCN × 14 days	OD 20/25	4 months
	OS HM	Panuveitis	IM PCN × 3 weeks	OS 20/80	
7	OD 20/25	Papillitis	IV ceftriaxone × 14 days	OD 20/40	4 months
	OS 20/20	Papillitis	Doxycycline po × 21 days	OS 20/40	
8	OD 20/30	Anterior uveitis	IV PCN × 14 days	OD 20/40	4 months
	OS 20/30	Anterior uveitis		OS 20/25	
9	OD 20/30	Panuveitis	IV PCN × 14 days	OD 20/30	1 months
	OS 20/30	Panuveitis		OS 20/40	
10	OD 20/150	Panuveitis	IV PCN × 14 days	OD 20/70	1 months
	OS HM	Panuveitis		OS 8/200	
11	OD 20/400	Posterior uveitis	IV PCN × 14 days	OD 20/150	7 months
	OS 20/200	Posterior uveitis		OS 20/150	
12	OD 20/25	Posterior uveitis	IV PCN incomplete	OD NA	Lost to FU
	OS HM	Posterior uveitis, CN III, VII palsy		OS NA	
13	OD prosthesis	Anterior uveitis	IV PCN × 12 days	OD prosthesis	1 months
	OS 20/25			OS 20/40	
14	OD 20/30	None	IV PCN × 10 days	OD 20/25	3 years
	OS 8 ft/200	Panuveitis		OS 20/400	
15	OD CF	Posterior uveitis	Lost to FU	OD NA	Lost to FU
	OS HM	Posterior uveitis		OS NA	
16	OD NLP	Pale disc	IV PCN × 14 days	OD NLP	12 months
	OS 20/60	Papillitis		OS 20/40	

*VA* visual acuity, *FU* follow-up, *LP* light perception, *HM* hand motion, *NLP* no light perception, *PCN*, penicillin

Our study also shows significantly higher serum RPR titers found in HIV-positive patients (range 1:64–1:16,348). Although it was previously suggested that the complex interaction of syphilis and HIV infection may cause non-treponemal tests such as VDRL or RPR serology to become falsely positive or negative, in general it has been suggested that non-treponemal test titers may be higher among HIV-positive patients than among HIV-negative patients [15–23]. Nevertheless, the high serum RPR titers in our HIV-positive patients are unlikely to be falsely positive because the diagnosis of ocular syphilis of all of our patients was supported by positive serum treponemal tests. In addition, the patients’ high RPR titers decreased following IV penicillin treatment in those who were retested, and ocular inflammation and vision improved after treatment. While HIV is considered to be one of the important causes of a false-positive reaction to serum non-treponemal test, in HIV-positive patients, a positive non-treponemal test requires careful evaluation by pairing it with a treponemal test when ocular syphilis is clinically suspected and high baseline serum RPR titers are present. The non-treponemal test is also useful to monitor the response to treatment in addition to ocular examination.

Our study also demonstrates frequent positive CSF FTA-ABS or VDRL test results in HIV-positive patients (60 % in HIV-positive vs 16 % in HIV-negative); however, due to the low numbers in the study, this was not found to be statistically significant. In our study, all patients except for one underwent lumbar puncture for CSF analysis. While CDC recommends CSF examination only if a patient has either neurological or ophthalmic signs or symptoms, evidence of active tertiary syphilis, or serologic treatment failure, whether or not they are infected with HIV, the criteria for CSF exam is still controversial [24]. Other guidelines still propose LP in HIV-positive patients with RPR titers of ≥1:32 or a CD4 cell count of <350 cells/ml [25, 26]. Others recommend that any HIV patient with syphilis should have an LP regardless of the presence of neurologic symptoms [26]. In the setting of a positive syphilis serology test, CSF abnormalities such as elevated CSF protein or white blood cell count or positive CSF VDRL or FTA-ABS results are useful to diagnose neurosyphilis. In our study, 6 of 10 HIV patients were positive for CSF VDRL or FTA-ABS. Since false-positive CSF VDRL or FTA-ABS values are very rare, these findings suggest the involvement of the central nervous system by syphilis in patients with HIV. However, none of these patients showed additional neurologic manifestations on clinical and radiographic exam. These findings suggest that ocular syphilis can present as a solitary manifestation of neurosyphilis without other overt neurologic impairments. On the other hand, our study showed that CSF exam of four out of five HIV-negative patients showed negative CSF VDRL or FTA-ABS but elevated CSF protein or CSF WBC count in three out of four patients. These findings in the HIV-negative group also may suggest CNS involvement or at least a compromised blood brain barrier because CSF VDRL or FTA-ABS is considered to be highly specific yet low sensitivity tests (22 to 69 %) and elevated CSF protein or WBC count were not confounded by other conditions such as HIV [11]. In this context, regardless of HIV status, our study supports the need for CSF analyses and parenteral penicillin treatment for ocular syphilis patients. Also, these study results support the recent clinical advisory from CDC on ocular syphilis. The CDC reported 24 newly diagnosed ocular syphilis cases from California and Washington since December 2014, recommended lumbar puncture with CSF examination in any patients with syphilis and ocular complaint, and recommended management of ocular syphilis according to the treatment recommendations for neurosyphilis [27]. Current guidelines from CDC on the treatment of neurosyphilis are either intravenous aqueous crystalline penicillin G or intramuscular procaine penicillin with probenecid for 10–14 days [24, 27]. As an alternative agent, the United Kingdom (UK) recommended oral doxycycline 400 mg daily for 28 days in patients who are allergic to penicillin or patients who refuse inpatient intravenous therapy [28].

Upon diagnosis of ocular syphilis in our HIV-positive patients, HIV-1 viral load ranged from 24,561 to >5,000,000 copies/ml (median 206,887; mean 1,008,779 ± 17,306) and this was higher than what was previously reported in neurosyphilis (median 67,450; range 11,900–113,000) or in ocular syphilis (mean 145,087.6 ± 166; range 30,321–>500,000) [29–31]. In our study, CD4 cell count typically ranged from 127 to 535 (mean 237 ± 142). Although syphilis is not an opportunistic infection such as cryptosporidiosis, cryptococcosis, pneumocystis carinii, or cytomegalovirus (50 < CD4 < 100 cells/ml) and mycobacterium avium complex (CD4 < 50 cells/ml), our study suggests that high HIV-1 viral load and relatively compromised immune status in HIV patients may play a role in ocular manifestation of syphilis [31].

## Conclusions

In the new era of reemergence of ocular syphilis associated with positive HIV status, we report that ocular syphilis in HIV patients is associated with more severe or diffuse forms of ocular inflammation such as panuveitis. Some features may be more common in HIV-positive patients compared to HIV-negative, such as disc swelling. These patients may have higher RPR titers and are more likely to demonstrate CSF VDRL or FTA-ABS positivity then HIV-negative patients. This emphasizes the importance of testing all new cases of ocular syphilis for HIV.
